# A novel histopathological grading system for ganglioglioma

**DOI:** 10.25122/jml-2021-0054

**Published:** 2021

**Authors:** Antonia Carmen Lisievici, Diana Pasov, Tiberiu-Augustin Georgescu, Mihai Gheorghe Lisievici, Maria Sajin

**Affiliations:** 1. Department of Pathology, Carol Davila University of Medicine and Pharmacy, Bucharest, Romania; 2. Department of Pathology, Bagdasar-Arseni Emergency Clinical Hospital, Bucharest, Romania; 3. Department of Pathology, National Institute for Mother and Child Health Alessandrescu-Rusescu, Bucharest, Romania; 4. Department of Pathology, Emergency University Hospital Bucharest, Bucharest, Romania

**Keywords:** ganglioglioma, anaplastic ganglioglioma, grading ganglioglioma

## Abstract

Gangliogliomas are central nervous system tumors located in the temporal lobe of young patients, frequently associated with epilepsy. In this paper, we propose a grading system based solely on histopathological criteria. We reevaluated all cases of ganglioglioma, atypical ganglioglioma, and anaplastic ganglioglioma diagnosed between 2011 and 2020 in the Pathology Department of the Emergency Clinical Hospital Bagdasar-Arseni, based on the type of glial mitoses, the number of neuronal and glial mitoses, presence of necrosis, microvascular proliferation, eosinophilic granular bodies, hypercellularity, presence and disposition of inflammatory infiltrate and atypical pleomorphism. Based on the proposed grading system, a score of 0–4 corresponded to a benign ganglioglioma, 5–9 to an atypical ganglioglioma, and 10–18 to an anaplastic ganglioglioma. The survival rates were 90% for benign ganglioglioma, 71.43% for atypical ganglioglioma, and 62.54% for anaplastic ganglioglioma. One case of benign ganglioglioma underwent a malignant transformation into anaplastic ganglioglioma, and recurrences were noticed in 28.57% of atypical ganglioglioma cases and 30.7% of all anaplastic gangliogliomas. The presence of rare glial mitoses and hypercellularity was correlated with mortality in cases of atypical ganglioglioma. We believe this histopathological scoring system could be used as a three-tier system to identify atypical ganglioglioma cases that are bound to have an aggressive course of evolution and require close follow-up. The other option would be to convert it to a two-tier grading system that can separate low-grade gangliogliomas from high-grade ones. The latter category can encompass both atypical and anaplastic ganglioglioma due to the high mortality of both entities.

## Introduction

Gangliogliomas are tumors of the central nervous system composed of a dual neoplastic population and showing a predilection for the temporal lobe [1, 2]. Neoplastic proliferation is constituted of both dysplastic neurons and neoplastic glial cells [3]. The latter frequently imparts an aspect of pilocytic astrocytoma, but an infiltrative glioma can rarely be part of the neoplastic proliferation, making anaplastic ganglioglioma the correct diagnosis [1, 4, 5]. The latest edition of the World Health Organization (WHO) classification of central nervous system tumors only recognizes a benign (grade I) ganglioglioma and a malignant variant, called anaplastic ganglioglioma (grade III). Nonetheless, the book's authors mention that there is a necessity for an intermediate, grade II ganglioglioma, but no histological criteria have been established [6].

In this paper, we propose a novel histopathological grading system for gangliogliomas, which also includes a grade II ganglioglioma. A number of 50 patients have been included in this study, which aims to correlate the histopathological features of gangliogliomas with the overall survival rates. The median follow-up period was 60.08 months. To our knowledge, there are no scientific studies in the literature encompassing benign, atypical, and anaplastic gangliogliomas aiming to implement a histological grading system. A recent publication has evaluated low-grade gangliogliomas in the adult population, focusing on epidemiological and clinical data, but less emphasis was given to histology [7].

## Material and Methods

We have conducted a retrospective study, analyzing all ganglioglioma cases, benign and malignant, diagnosed during a period of 9 years (2011–2020) in the Pathology Department of the Emergency Clinical Hospital Bagdasar-Arseni. Hematoxylin-eosin and Van-Gieson stained slides have been thoroughly reviewed by the authors, excluding those cases where the diagnosis was questionable and those cases where ancillary studies did not confirm the diagnosis. The clinical chart was retrieved from the hospital archive to obtain relevant clinical data (comorbidities, symptoms, presence of epileptic seizures, and recurrences). Imagistic features were also acquired, including dimensions of lesions and the presence of cystic or solid areas.

The histological features used to conceive the grading algorithm included elements of the glial component: type of glial proliferation (pilocytic/infiltrative glioma), presence of eosinophilic granular bodies (Rosenthal fibers), presence of microvascular proliferation, number of mitoses, presence of necrosis, degree of cellularity and cellular monomorphism/pleomorphism. The neuronal component was evaluated for the presence of mitoses, type of inflammatory infiltrate (perivascular/diffuse/absent), and whether it was predominant or represented only a small component of the tumoral bulk. According to the scoring algorithm pictured in Table 1, we have assigned a score of 1–4 to grade I ganglioglioma, a score of 5–9 to atypical (grade II) gangliogliomas, and a score of 10–18 to anaplastic gangliogliomas.

**Table 1.** Histopathological grading system for gangliogliomas.

Score	0	1	2
**Glial component**			
**Subtype**	Pilocytic astrocytoma	Atypical glial proliferation	Diffusely infiltrative glioma
**Cellularity**	Mild	Moderate	High
**Granular eosinophilic bodies**	Present	Absent	-
**Microvascular proliferation**	Pilocytic/glomeruloid type	Absent	True malignant microvascular proliferation
**Mitoses**	Absent	Rare	“Striking”
**Necrosis**	Absent	Focal	Diffuse
**Atypical cellular monomorphism**	Absent	-	Present
**Neuronal component**			
**Quantitative assessment**	Neuronal component prevails	Rare dysmorphic bi- or multinucleated neurons	-
**Mitoses**	Absent	Rare	“Striking”
**Inflammatory infiltrate**	Perivascular and diffuse	perivascular	Absent

## Results

Analyzing the clinical data, we observed that patients’ age varied from 3 to 77 years old, with a median of 32.5 and an average of 36.94 years. The cases were distributed fairly equally between the two genders, with a slight predilection for the male gender (56% of all cases). The addressability of the patients originated both from the urban and rural environment, with a slight increase in patients from the former (54.05%). Interestingly, 60% of all tumors occurred in the right hemisphere, while only 34% were found in the left hemisphere and 16% on the median line. Gangliogliomas most frequently involved the temporal lobe (32%), closely followed by the frontal lobe (20%), parietal lobe (16%), and posterior fossa (14%). A minority of cases involved multiple lobes (12%), while the occipital lobe (4%) and the conus medullaris (2%) were only exceptionally affected. The infiltration of multiple lobes or central nervous systems by anaplastic gangliogliomas has been previously reported in the literature [8].

**Figure 1.**
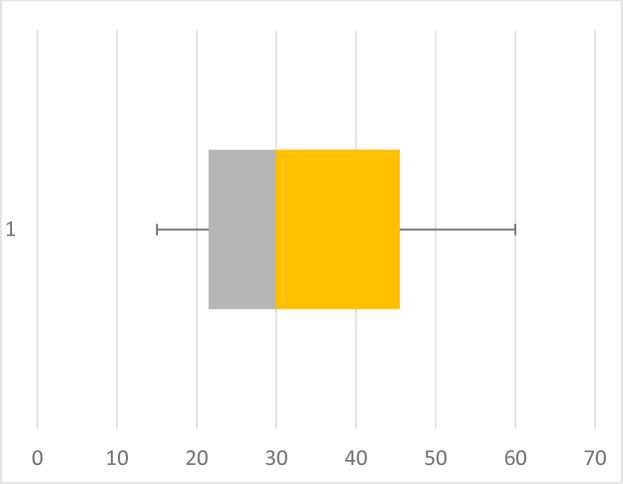
Size variation of grade I gangliogliomas.

**Figure 2.**
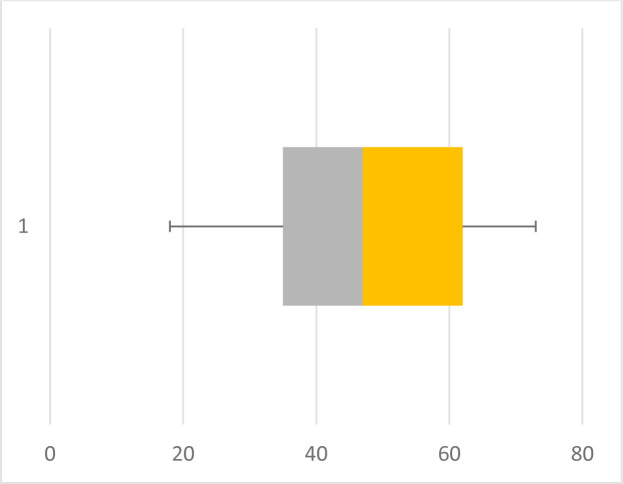
Size variation of grade III gangliogliomas.

**Figure 3.**
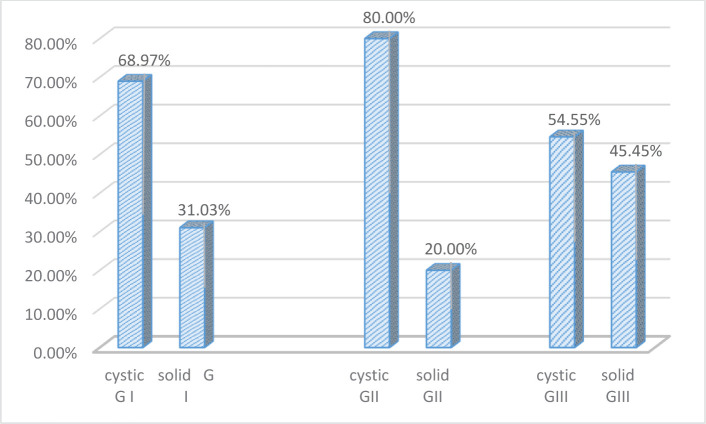
Imagistic aspects of grade 1, grade 2 and grade 3 gangliogliomas.

Regarding the imagistic aspects of the lesions, anaplastic gangliogliomas had significantly larger sizes, with a median of 47 mm, while grade I gangliogliomas had a median of 30 mm. A short comparison of the two entities can be observed in the box plot pictured in Figure 1 and Figure 2. The majority of cases (66%) featured a cystic component, while 34% presented as a solid tumor with an inhomogeneous appearance. Out of the 30 assessed grade I gangliogliomas, 68.97% featured a cystic component, while only 54.55% of all 13 anaplastic gangliogliomas had a cystic component. The ones classified as grade II gangliogliomas were the ones with the lowest percentage of cases composed only of a solid tumor (20%). All imagistic aspects have been summarized in Figure 3.

Epilepsy was present in 58% of all reviewed cases; 58.62% of all grade I ganglioglioma, 70% of all grade II gangliogliomas, and 45.45% of all anaplastic gangliogliomas associated epileptic seizures.

Using the previously described algorithm, we classified the results as follows: 30 cases of grade I ganglioglioma, 7 cases of grade II/atypical ganglioglioma, and 13 cases of anaplastic ganglioglioma.

Out of the 30 cases of grade I ganglioglioma, 13.3% had recurrences, and 3.33% suffered malignant transformation into an anaplastic ganglioglioma. In terms of mortality, the survival rate after a 2-year follow-up was 90%, while 6.66% died of disease, and 3.33% succumbed to perioperative mortality. The percentage of patients who died of disease was strongly correlated with the presence of recurrences, while perioperative mortality was encountered in those patients with atypical tumor location (e.g., retrobulbar). Although different studies from the literature have suggested that a fibrillary astrocytoma was more frequently encountered as a glial component, in our experience, a pilocytic astrocytoma was significantly more common [9, 10]. Additionally, fibrillary astrocytoma is no longer an entity recognized by the WHO classification of central nervous system tumors [6]. One particular case included in our study featured imagistic aspects suggestive of a dysembrioplastic neuroepithelial tumor.

The atypical ganglioglioma poll of cases, although reduced in number, provides unexpected information. Most cases evaluated in this study can be fairly easily evaluated as benign or anaplastic gangliogliomas. However, there exists a small percentage of cases that fall short of the normal criteria for the two mentioned entities, which can be viewed as two ends of a spectrum. The algorithm developed in this study aims to identify precisely those cases that might have aggressive behavior but do not meet the criteria for anaplastic gangliogliomas. The survival rate after 2 years for those cases that achieved a score between 5–9 was 28.57%. The death of disease rate was 71.43%, and even those who survived the 2-year follow-up suffered greatly from the morbidities caused by the recurrences, in a proportion of 100%.

The degree of cellularity was increased in these cases, with 72.43% of them featuring hypercellularity and 28.57% showing only moderate cellularity. Also, 57.14% of all atypical gangliogliomas and 60% of those who died of disease had a glial component featuring histopathological features of an atypical glial proliferation, which could not be classified as diffusely infiltrative glioma. No neuronal mitoses have been identified in either of these cases, similar to those cases of grade I ganglioglioma. 100% of those cases evaluated as grade II ganglioglioma had an inflammatory infiltrate located exclusively perivascularly. Regarding microvascular proliferation, 80% of cases in which the patients have died of disease had neither pilocytic nor malignant microvascular proliferation types. Rare glial mitoses have been identified in 100% of cases in which the patient died of disease. The presence of necrosis did not correlate with the survival rate, as only 28.57% of all atypical gangliogliomas featured focal or diffuse necrosis. Eosinophilic granular bodies were present in 85.71% of all grade II ganglioglioma cases, attesting to the slow development of the tumor.

Patients with anaplastic gangliogliomas had a survival rate at 2 years of 38.46%, and 30.7% of patients developed recurrences along with the evolution of the disease. The incidence of recurrences was similar between the cases in which the patient died of disease and those in which the patients were still alive at the time of the present study. Malignant microvascular proliferation, similar to that encountered in glioblastomas, was present in 84.61% of cases, while neuronal mitoses were present in 76.92% and glial mitoses in 100% of all anaplastic gangliogliomas. Nonetheless, striking mitotic activity was present in the glial component in 30.76% of cases. Necrosis was present in 92.30% of cases, although only 23.07% of cases featured diffuse areas of necrosis. It is also worth acknowledging that 76.92% of cases did not feature any eosinophilic bodies.

## Discussion

As stated above, grade I ganglioglioma is usually easily identified without the mentioned algorithm. It does not feature necrosis, mitosis, hypercellularity, and glial proliferation usually has features of pilocytic astrocytoma. The presence of recurrences was correlated with the incidence of death in these patients. Similarly, cases of anaplastic ganglioglioma featured an anaplastic glial component, and mitoses were present in both components (glial and neuronal). The mortality rate was significantly higher compared to the benign ganglioglioma cases.

Surprisingly, the grade II gangliogliomas also featured a high mortality rate (71.43%), similar to that encountered in anaplastic ganglioglioma. Thus, one could raise the question – why implement a three-tiered grading system if the prognostic between grade II and grade III would be similar? The authors consider that a two-tiered system of grading, like the one being applied right now, will undervalue many cases that will evolve aggressively due to relatively bland histology. Lowering the threshold for diagnosis of anaplastic ganglioglioma could be a solution, but that should also be done according to a scoring system as well. Further studies should be conducted in order to verify the reproducibility of this algorithm. Although the experts are still struggling to find histopathological criteria to identify those cases of grade II ganglioglioma at this moment, the recently published papers are making significant progress in terms of technology, namely distinguishing glioneuronal tumors from focal cortical dysplasia, by machine learning [11].

The recent literature does not provide much information about the prognosis of atypical (grade II) ganglioglioma [12, 13]. Nonetheless, Čupić *et al.* reported a case of atypical ganglioglioma, which has progressed into a glioblastoma, confirming the unpredictable nature of these tumors [14]. Although the cases classified as grade II ganglioglioma have had a high mortality rate, previous studies have reported different outcomes, reflecting the ambiguity of the previous classification and the poor interobserver reproducibility. Majores *et al.* reported a survival rate of 79% in atypical gangliogliomas, compared to 53% in those cases of anaplastic ganglioglioma [12, 15, 16].

Another approach proposed in the recent literature was to attribute the term “atypical” to those cases of ganglioglioma that have unusual clinical or imagistic features, either an atypical location or an infiltrative aspect [17–19]. This approach seems to have correlated with the poor evolution and recurrence of the disease [20]. Our study does not completely endorse this data since the average size of those cases that ended in death for the patient is 40.8%, while the average size of those tumors of patients who are still alive today is 35.84%. Also, recurrences were present in 17% of patients who died of disease and 20.50% of those who survived, so there is no significant difference. Nonetheless, the frontal location of the tumor was associated with an unusual evolution of the disease, independent of the histological grade of the tumor: 50% of patients died of disease, and 33.30% suffered recurrences. In comparison, only 18% of patients with tumors located in the temporal lobe dies of disease or had recurrences. Other studies that analyzed the neuroradiologic features of gangliogliomas have also observed features of dysembrioplastic neuroepithelial tumor in 75% of cases [21]. In contrast, our study only observed these aspects in a single case of benign ganglioglioma.

## Conclusion

The proposed algorithm can be applied in one of two ways. One can endorse the term atypical ganglioglioma (grade II), acknowledging the high mortality included in this group of cases requiring close follow-up and, probably, even a more aggressive treatment to contain the disease. The alternative would represent a two-tier grading system, in low-grade and high-grade ganglioglioma, the latter encompassing both atypical and anaplastic gangliogliomas. This variant would probably have a better correlation with the clinical behavior of the tumor and might be more easily accepted by pathologists around the world. Nonetheless, this variant still requires an algorithm that helps one identify those cases that might have a poor outcome.

Regarding the histological and clinical aspects of the tumor, one should pay careful attention to the presence of mitoses in the glial proliferation of all ganglioglioma cases, to the hypercellularity, presence of an inflammatory infiltrate with a unique perivascular disposition, and to the frontal location of the tumor.

## Acknowledgments

### Conflict of interest

The authors declare that there is no conflict of interest.
